# Identifying the Long-Term Role of Inducible Nitric Oxide Synthase after Contusive Spinal Cord Injury Using a Transgenic Mouse Model

**DOI:** 10.3390/ijms18020245

**Published:** 2017-01-25

**Authors:** Dominic M. Maggio, Amanpreet Singh, J. Bryan Iorgulescu, Drew H. Bleicher, Mousumi Ghosh, Michael M. Lopez, Luis M. Tuesta, Govinder Flora, W. Dalton Dietrich, Damien D. Pearse

**Affiliations:** 1 The Miami Project to Cure Paralysis, University of Miami Miller School of Medicine, Miami, FL 33136, USA; dominic.maggio@nih.gov (D.M.M.); amanpu@gmail.com (A.S.); jbi2001@med.cornell.edu (J.B.I.); drew.bleicher@jhsmiami.org (D.H.B.); mghosh@med.miami.edu (M.G.); Michael.Lopez@mch.com (M.M.L.); luis_tuesta@hms.harvard.edu (L.M.T.); gjsflora@gmail.com (G.F.); ddietrich@miami.edu (W.D.D.); 2 Department of Neurological Surgery, University of Virginia School of Medicine, Charlottesville, VA 22908, USA; 3 Surgical Neurology Branch, National Institute of Neurological Disorders and Stroke, National Institute of Heath, Bethesda, MD 20824, USA; 4 Department of Pathology, Brigham and Women’s Hospital, Harvard Medical School, Boston, MA 02115, USA; 5 Department of Neurological Surgery, University of Miami Miller School of Medicine, Miami, FL 33136, USA; 6 Department of Genetics, Harvard Medical School, Boston, MA 02115, USA; 7 The Neuroscience Program, University of Miami Miller School of Medicine, Miami, FL 33136, USA; 8 The Interdisciplinary Stem Cell Institute, University of Miami Miller School of Medicine, Miami, FL 33136, USA; 9 Department of Neurology, University of Miami Miller School of Medicine, Miami, FL 33136, USA; 10 Department of Cell Biology and Anatomy, University of Miami Miller School of Medicine, Miami, FL 33136, USA; 11 Bruce W. Carter Department of Veterans Affairs Medical Center, Miami, FL 33136, USA

**Keywords:** oxidative stress, neuroprotection, angiogenesis, inducible nitric oxide synthase, knockout, axon, function

## Abstract

Inducible nitric oxide synthase (iNOS) is a potent mediator of oxidative stress during neuroinflammation triggered by neurotrauma or neurodegeneration. We previously demonstrated that acute iNOS inhibition attenuated iNOS levels and promoted neuroprotection and functional recovery after spinal cord injury (SCI). The present study investigated the effects of chronic iNOS ablation after SCI using *inos*-null mice. *iNOS^−/−^* knockout and wild-type (WT) control mice underwent a moderate thoracic (T8) contusive SCI. Locomotor function was assessed weekly, using the Basso Mouse Scale (BMS), and at the endpoint (six weeks), by footprint analysis. At the endpoint, the volume of preserved white and gray matter, as well as the number of dorsal column axons and perilesional blood vessels rostral to the injury, were quantified. At weeks two and three after SCI, *iNOS^−/−^* mice exhibited a significant locomotor improvement compared to WT controls, although a sustained improvement was not observed during later weeks. At the endpoint, *iNOS^−/−^* mice showed significantly less preserved white and gray matter, as well as fewer dorsal column axons and perilesional blood vessels, compared to WT controls. While short-term antagonism of iNOS provides histological and functional benefits, its long-term ablation after SCI may be deleterious, blocking protective or reparative processes important for angiogenesis and tissue preservation.

## 1. Introduction

Every year, approximately 16,000 Americans suffer a spinal cord injury (SCI) [1], leaving them with a permanently diminished ambulatory ability and decreased quality of life, aspects which remain a major focus for restorative approaches [2,3]. Pathologic processes involved in cell death during the post-impact period have been targeted by various approaches, which aim to improve the survival of potentially salvageable spinal cord tissue [4]. Tissue destruction following SCI occurs in two phases. The first of these phases is the primary injury, which is characterized by cellular necrosis resulting from the mechanical impact of the SCI. The primary injury is followed by a phase of secondary injury, which lasts for a period of time between several days and weeks, in response to an abundance of cytotoxic molecules, an overzealous inflammatory response, and the activation of neural cell death signaling pathways [4]. Propagation of reactive oxygen and nitrogen species, such as the superoxide anion (O_2_^●−^), hydroxyl radical (^●^OH), nitric oxide (NO), and peroxynitrite (ONOO^−^), can drive secondary injury processes. These reactive species and downstream effectors can directly damage intracellular proteins and membrane phospholipids through oxidation, as well as activate redox-responsive transcription factors that promote inflammation [5,6,7,8,9]. Accordingly, therapeutic strategies for SCI that antagonize oxidative stress through the use of antioxidant compounds or agents that upregulate radical disproportionation enzymes, have demonstrated promising results in vivo [10,11,12,13,14,15,16].

NO is a diffusible product of nitric oxide synthase’s (NOS) conversion of l-arginine to l-citrulline. NO is involved in a wide variety of physiological processes, such as immune modulation, neurotransmission, hormone release, and vasodilation [17,18]. The NOS family is comprised of three isoforms: neuronal NOS (nNOS, type I), endothelial NOS (eNOS, type III), and inducible NOS (iNOS, type II). Whereas nNOS and eNOS are calcium-dependent, constitutively expressed enzymes, iNOS is calcium-independent and is largely expressed by cells of the innate immune system, in response to cell damage and pro-inflammatory cytokines [19]. Production of nitric oxide through iNOS is associated with higher and more persistently-elevated levels of NO, relative to NO production through other means [19]. When NO is produced in an environment of oxidative stress, such as following SCI, NO combines with the superoxide radical to form the highly reactive oxidizing agent, peroxynitrite. This molecule is highly apoptogenic due to its potent destruction of nucleic acids, as well as its ability to inactivate membrane lipoproteins and essential intracellular proteins [5,18,20]. Peroxynitrite may also be an important trigger of lipid peroxidation [21]. In models of neurological injury, studies have shown a similar spatial and temporal localization of 3-nitrotyrosine (3-NT), a marker for protein nitration, and 4-hydroxynonenal (4-HNE), which is used to identify lipid peroxidation following both traumatic brain injury [22,23] and SCI [24]. Peroxynitrite also antagonizes mitochondrial respiration [25] and induces strand breaks in DNA that can activate poly(ADP-ribose) polymerase (PARP) to trigger cell death [26].

To target iNOS after contusive SCI, we previously employed selective pharmacological inhibitors, such as 1400W, or *inos*-specific antisense oligonucleotides, the latter of which was more effective in acutely reducing iNOS expression and activity, limiting neutrophil infiltration and myeloperoxidase production, as well as decreasing blood-spinal cord barrier permeability, reactive astrogliosis, and neuronal cell death [12]. The acute perturbation of these processes through iNOS inhibition also translated into a significant improvement in locomotor recovery after SCI [27]. However, these investigations only involved a short-term suppression of iNOS, whereby the length of inhibition was limited to a period of days by the in vivo stability of the delivered oligonucleotides. Whether a longer term molecular inhibition of iNOS would provide greater efficacy was not examined. Although iNOS is involved in deleterious processes and tissue damage after SCI, both iNOS and its production of NO have also been implicated in the processes of wound healing and repair [28,29]. To begin to understand the optimal duration for iNOS antagonism after SCI, the present study employed *inos*-null mice, which exhibit a complete and permanent loss of this enzyme after SCI. Identifying the optimal duration of iNOS inhibition required for maximal efficacy after SCI, is an important question to address for eventual therapeutic consideration of this target as a neuroprotective approach.

## 2. Results

### 2.1. White and Gray Matter Preservation Was Significantly Less in Inducible Nitric Oxide Synthase (iNOS^−/−^) Mice after Spinal Cord Injury (SCI)

**Figure 1:**
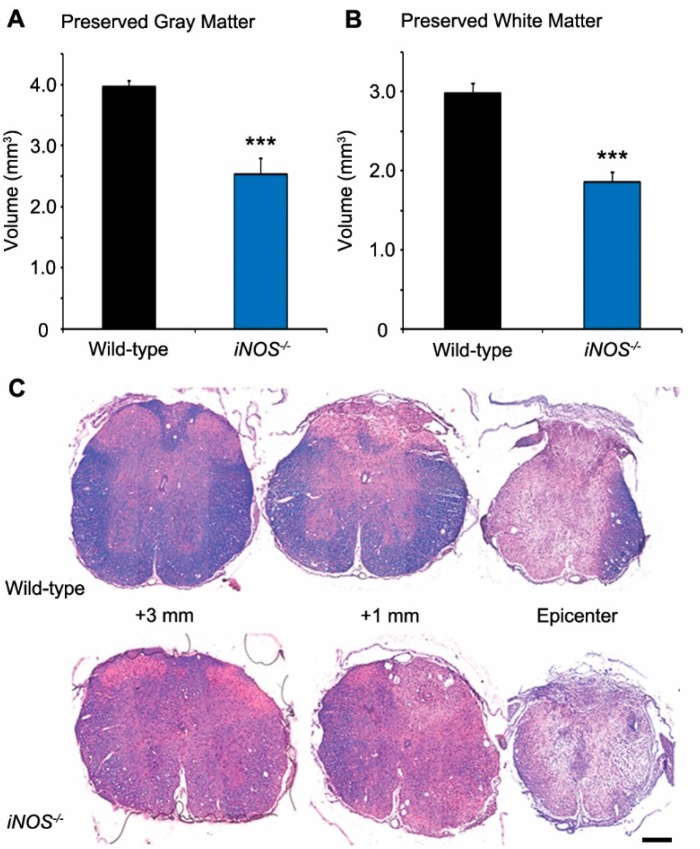
White and gray matter tissue preservation after spinal cord injury (SCI) was significantly less in inducible nitric oxide synthase (iNOS) knockout mice compared to wild-type (WT) controls. At week six, post-SCI, *iNOS^−/−^* and WT mice showed significant reductions in the volumes of healthy gray and white matter within the injured spinal cord segment. Compared to WT animals, *iNOS^−/−^* had smaller volumes of preserved white (**A**); and gray (**B**) matter after SCI; (**C**) Representative transverse sections stained with hematoxylin, eosin, and luxol fast blue from WT and *iNOS^−/−^* mouse spinal cord tissue at the injury epicenter, as well as 1 and 3 mm rostral. Statistical significance indicated at *** *p* < 0.001 versus injured WT controls. Scale bar = 150 µm.

Injury to the spinal cord produced a significant reduction in the volume of healthy-appearing gray matter at six weeks post-SCI. Compared to WT mice (3.97 ± 0.21 mm^3^), the volume of preserved gray matter within the injured thoracic spinal cord was significantly less in *iNOS^−/−^* mice after SCI (2.54 ± 0.13 mm^3^, a 37% decrease, *p* < 0.001; Figure 1A,C). Similarly, SCI led to a significant decrease in healthy-appearing white matter. In comparison to WT mice at six weeks post-SCI (2.98 ± 0.41 mm^3^), there was significantly less preserved white matter within the injured thoracic spinal cord of *iNOS^−/−^* mice (1.87 ± 0.12 mm^3^, a 38% reduction, *p* < 0.001; Figure 1B,C).

### 2.2. Fewer Dorsal Column Neurofilament (NF)^+^ Axon Profiles and Perilesional Blood Vessels Were Present Rostral to the Lesion in Inducible Nitric Oxide Synthase (iNOS^−/−^) Mice after Spinal Cord Injury (SCI)

**Figure 2:**
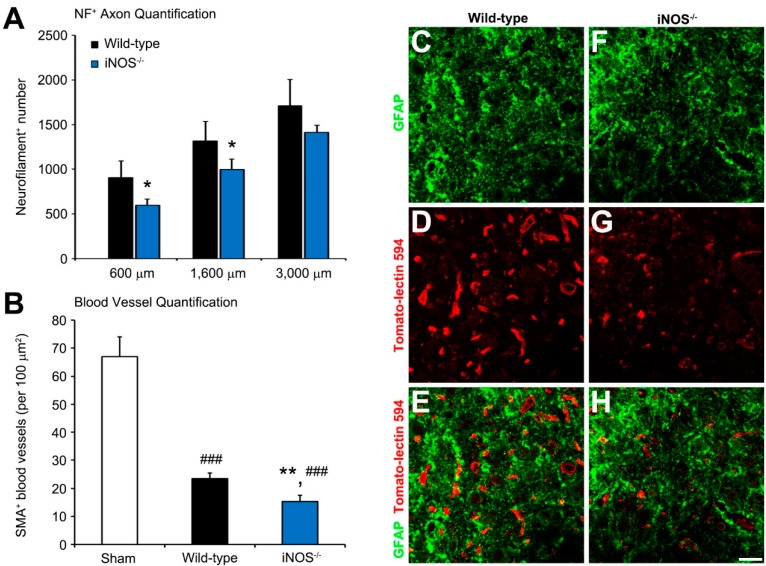
Numbers of dorsal column neurofilament (NF)^+^ axons and perilesional blood vessels within the spinal cord rostral to the spinal cord injury (SCI) were significantly fewer in inducible nitric oxide synthase (*iNOS*) knockout mice than wild-type (WT) controls. Counts of dorsal column axons at six weeks post-SCI within tissue sections at specific distances rostral to the injury showed that *iNOS^−/−^* mice exhibited fewer NF^+^ axons at 600, 1600, and 3000 µm, rostral to the SCI epicenter compared to WT controls (**A**); At six weeks post-SCI, *iNOS^−/−^* mice also displayed 35% fewer perilesional smooth muscle action (SMA)^+^ blood vessels compared to WT controls (**B**); Both *iNOS^−/−^* and WT control groups demonstrated substantially fewer perilesional blood vessels (−77% and −65% respectively) when viewed against a comparable uninjured spinal cord region from sham controls. Representative micrograph images of similar regions of perilesional lateral white matter from the spinal cord rostral to the SCI of (**C**–**E**) WT and (**F**–**H**) *iNOS^−/−^* mice immunostained for glial fibrillary acidic protein (GFAP) and Tomato-lectin-594 were used to identify astrocytes and blood vessels, respectively. Significantly fewer blood vessels were identified within iNOS^−/−^ mice at six weeks after SCI. Statistical significance indicated at * *p* < 0.05 or ** *p* < 0.01 versus injured WT controls and ^###^ *p* < 0.001 versus sham controls. Scale bar = 25 µm.

The dorsal columns within the rostral, injured spinal cord of *iNOS^−/−^* mice contained significantly fewer NF^+^ axons six weeks post-SCI than observed in wild-type (WT) mice at 600 µm (34% fewer, 595 ± 71 axons vs. 905 ± 187 axons, *p* < 0.05), 1600 µm (32% fewer, 995 ± 120 axons vs. 1315 ± 219 axons, *p* < 0.05), and 3000 µm (21% fewer, 1410 ± 80 axons vs. 1710 ± 296 axons, *p* > 0.05), rostral to the center of the lesion (Figure 2A). These axonal pathways encompass the corticospinal and spinothalamic tracts, which are at the epicenter of the injury.

Because *iNOS* knockout may differentially affect vascular smooth muscle and endothelial physiology, perilesional vessels were assessed by both smooth muscle actin (SMA; denoting vascular smooth muscle cells) and Tomato-lectin-594 (denoting vascular endothelial cells) staining. At six weeks post-SCI, *iNOS^−/−^* mice also exhibited 35% fewer perilesional blood vessels (15 ± 2 vessels) compared to injured WT controls (23 ± 2 per 100 µm^2^, *p* < 0.01), as identified by labeled SMA^+^ profiles (Figure 2B). After SCI, both *iNOS^−/−^* and WT animals had less blood vessels within the perilesional white matter (−77% and −65% respectively, *p* < 0.001) compared to sham controls (67 ± 7 vessels per 100 µm^2^), as measured from the same spinal level. Rostral cord sections immunostained for GFAP and Tomato-lectin-594 of the perilesional lateral white matter showed a similar result, with significantly more labeled blood vessels being present in WT controls (Figure 2C–E) than within the iNOS^−/−^ knockout specimens (Figure 2F–H).

### 2.3. Inducible Nitric Oxide Synthase (iNOS^−/−^) Mice Showed an Acute Improvement in Functional Recovery over Wild-Type (WT) Controls after Spinal Cord Injury (SCI) That Did Not Persist Long-Term

**Figure 3:**
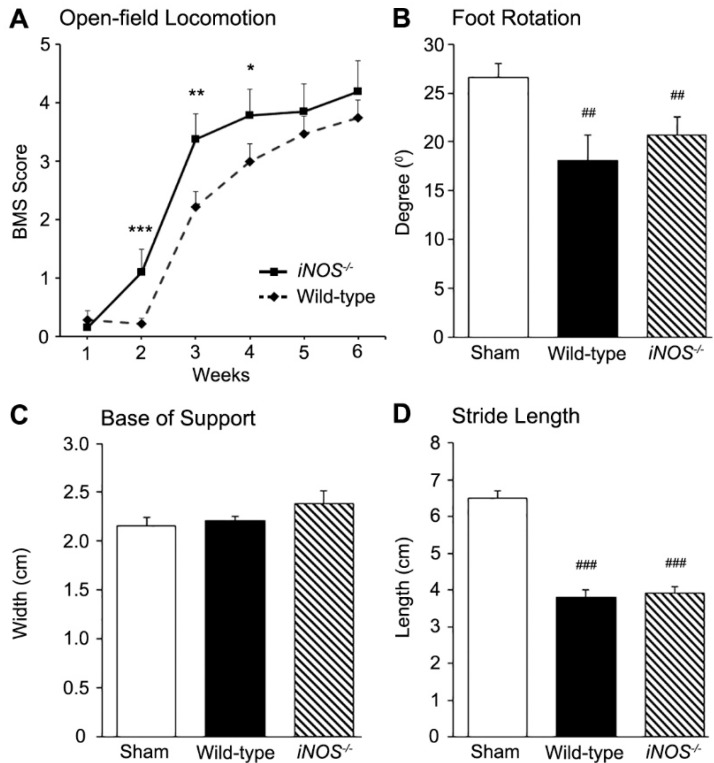
The knockout of inducible nitric oxide synthase (iNOS) significantly improved locomotor functional recovery acutely within the first month post-spinal cord injury (SCI), but did not provide a persistent locomotor benefit. The *iNOS^−/−^* knockout mice exhibited a more rapid and significant improvement in open field locomotion at week two than the wild-type (WT) controls (**A**); which remained significant at weeks three and four. However, at six weeks post-SCI, the difference in open-field locomotor performance between *iNOS^−/−^* and WT was not significant. Similarly, at the endpoint, there was no benefit of iNOS knockout over WT controls, in the degree of foot rotation (**B**); base of support (**C**); or average stride length (**D**), as measured by footprint analysis. Statistical significance indicated at * *p* < 0.05, ** *p* < 0.01, or *** *p* < 0.001 versus injured WT controls and ^##^ *p* < 0.01 or ^###^ *p* < 0.001 versus sham controls.

Following thoracic spinal cord contusion, complete flaccid paralysis of the hind limbs (BMS < 0.5) was evident for the week following SCI in both WT and *iNOS^−/−^* mice. During the second, third, and fourth weeks post-SCI, *iNOS^−/−^* mice exhibited an improved recovery, with significantly higher BMS scores than WT controls (week two: WT 0.2 ± 0.1 vs. *iNOS^−/−^* 1.1 ± 0.4, *p* < 0.001; week three: WT 2.2 ± 0.2 vs. *iNOS^−/−^* 3.4 ± 0.4, *p* < 0.01; and week four: WT 3.5 ± 0.3 vs. *iNOS^−/−^* 3.9 ± 0.5, *p* < 0.01; Figure 3A). However, BMS scores at later points in time, from five weeks post-SCI, showed no significant improvement in functional recovery between *iNOS^−/−^* and WT mice. Footprint analysis at six weeks post-SCI showed no differences in foot positioning and placement between *iNOS^−/−^* and WT mice, with no changes in foot rotation (20.7° ± 2.7° vs. 18.1° ± 4.1°, respectively; Figure 3B), base of support (2.4 ± 0.2 cm vs. 2.2 ± 0.1 cm, respectively; Figure 3C), or average stride length (3.9 ± 0.2 cm vs. 3.8 ± 0.3 cm, respectively; Figure 3D).

## 3. Discussion

The current investigation sought to build upon earlier work by both our group and others, demonstrating the therapeutic potential of targeting inducible nitric oxide synthase (iNOS) after spinal cord injury (SCI) [12,27,30,31,32] with the goal of understanding the pathological and functional consequences of longer term, complete ablation of iNOS using a transgenic mouse approach. At six weeks post-SCI, *iNOS^−/−^* mice exhibited less preserved white and gray matter, as well as fewer dorsal column axons and perilesional blood vessels rostral to the lesion, than wild-type (WT) controls, indicating a detrimental effect of long-term iNOS removal on injury histopathology and tissue repair.

Acutely after SCI, within the first few weeks, *iNOS^−/−^* mice exhibited significantly better functional recovery than WT controls, in accordance with prior findings [31]. However, at later time points, through the sixth week post-SCI, no significant differences in Basso Mouse Scale (BMS) scores or footprint analysis between groups were observed, although injured *iNOS^−/−^* mice did exhibit significantly worse histopathology than WT controls. While acute antagonism of iNOS with pharmacological [12,30,32], molecular [12,27], or transgenic [31] approaches, has demonstrated beneficial impacts after SCI, there is evidence from other neurological disease and injury paradigms, including traumatic brain injury (TBI), nerve injury, and experimental autoimmune encephalitis (EAE), that the complete ablation of iNOS, particularly for longer durations, can be deleterious. In experimental TBI, *iNOS^−/−^* mice exhibited significantly greater memory latency and markedly worse performance on the Morris water maze task at 20 days after injury, when compared to the WT injured controls [33]. Comparison of uninjured *iNOS^−/−^* and WT mice revealed no inherent differences in these outcomes. In a nerve injury paradigm, *iNOS^−/−^* mice exhibited retarded Wallerian degeneration, with subsequent delayed regeneration that was characterized by fewer distal regenerated myelinated fibers and less reinnervation of muscle endplates at six weeks after injury [34]. The slowing of these processes in *iNOS^−/−^* mice also led to a delay in the manifestation of neuropathic pain, but they showed slower long-term resolution of pain compared to WT controls. Although iNOS inhibitors have shown some efficacy in slowing disease in EAE, a model for Multiple Sclerosis (MS), induction of EAE in *iNOS^−/−^* mice led to a higher incidence and severity of disease, when compared to WT mice, that was characterized by an inability of *iNOS^−/−^* mice to undergo disease remission [35,36,37,38]. Long-term treatment of EAE in *iNOS^+/+^* mice with iNOS inhibitors has resulted in a similar exacerbation of deficits [39]. Subsequent studies have suggested that iNOS expression may help to keep CD4^+^ T-helper lymphocyte responses in check during chronic inflammation, whereby nitric oxide (NO) prevents interleukin-12 synthesis, type 1 T-helper proliferation, and excessive pro-inflammatory cytokine production, including that of interferon γ and tumor necrosis factor α (TNF-α) [40,41,42,43,44].

NO plays a central role in a diversity of physiological processes in the CNS, and its actions on a variety of cellular constituents can alter deleterious injury-related signaling, including its *S*-nitrosylation of *N*-Methyl-d-Aspartate (NMDA) receptors to downregulate their activity and prevent excitotoxicity, or of caspases to inhibit the apoptotic cell death cascade [45,46,47]. NO is also a potent stimulator of guanylate cyclase activity, raising levels of guanine monophosphate (cyclic GMP) that results in the activation of protein kinase G (PKG), which can promote the enhancement of the anti-apoptotic B-cell lymphoma 2 (Bcl-2) family of proteins, prevent cytochrome c release, and stimulate vascular endothelial growth factor (VEGF)-dependent angiogenesis [48,49,50,51]. Interestingly, although the expression levels of all three NOS isoforms increase acutely after SCI, at the later times in which we observed a lessened benefit of iNOS ablation, nNOS expression has been shown to fall significantly [7,30]. Evidence for the importance of basal neuronal NOS (nNOS) activity can be seen in studies of nNOS knockout mice following sciatic nerve transection. A deficiency in nNOS, particularly within spinal cord neurons, resulted in substantial interneuron and motoneuron degeneration, delayed motor and sensory recovery, abnormal Wallerian degeneration, and perturbed neurite pruning [52,53,54,55,56]. It has been demonstrated that NO produced by nNOS in response to peripheral nerve injury is needed as a signal for axonal damage, acting through hypoxia-inducible factor-1 and erythropoietin to neighboring Schwann cells to alter their function such that further axonal degeneration is prevented via their secretion of supportive neurotrophic factors [57,58].

Constitutive basal production of NO by nNOS and endothelial NOS (eNOS) are thought to contribute to physiologic suppression of iNOS in the non-injured spinal cord [30,59,60,61]. Although expression of iNOS in the uninjured spinal cord, absent of inflammation and trauma, has not reliably been observed [62,63,64], Hervera and colleagues investigated transgenic iNOS deficient mice for differences in sham- and post-injury spinal cord expression of nNOS and eNOS, compared to WT mice [56]. Post-injury mRNA and protein levels of nNOS were notably decreased in the iNOS-deficient mouse, without a corresponding change in eNOS levels. This highlights the possibility of iNOS production in the acute phase of SCI, which acts in a positive feedback manner to temporally increase levels of nNOS, possibly contributing to peroxynitrite production and secondary injury. No differences in nNOS and eNOS mRNA and protein expression were seen across sham-injury WT and iNOS^−/−^ groups. This limits the possibility of differential production of iNOS in the pre-neurologic injury setting contributing to histologic and functional differences post-SCI. Additionally, transgenic iNOS deficient mice are noted to have grossly identical spinal cord architecture to WT mice by H&E, Luxol fast blue, and microtubule-associated protein 2 (MAP2) immunohistochemistry, staining gray/white matter, myelin, and neurons, respectively [31,65].

Transgenic iNOS deficient mice are ideal models for investigating the functional and behavioral consequences of long-term iNOS suppression post-SCI, due to technical concerns associated with alternative methods of long-term iNOS attenuation or the level of iNOS inhibition achieved in the long-term. Long-term administration of anti-iNOS pharmacologic agents may be limited by their effectiveness to cross the blood-spinal cord-barrier (BSCB) at the chronic stage of SCI and their degree of iNOS inhibition compared to absolute ablation. Chronic silencing of iNOS through RNA interference is more difficult than use of a transgenic, due to the requirement for multiple repeated surgeries to permit direct spinal cord administration. Future studies attempting to further characterize the ideal iNOS suppression timeframe, however, might be best suited to the development of a mouse model capable of conditional iNOS attenuation, allowing knockout only during the post-SCI period, overcoming any pre-injury effects, and permitting timed control over the timing of the knockout, at acute or chronic stages of injury. Such a model is currently unavailable, but likely within the realm of current technological possibility.

Future studies should seek to investigate the differences in the cellular localization of iNOS after chronic SCI. Post-contusive SCI results in an influx of neutrophils, microglia, and other inflammatory mediators [66,67] known to express high levels of iNOS, which contribute to the formation of reactive oxygen species such as peroxynitrite, as mentioned previously [21,68,69,70]. Xu et al. [71], investigated iNOS immunoreactivity in the first two weeks after SCI and found iNOS localization in perilesional neurons, astrocytes, oligodendrocytes, endothelial cells, and ependymal cells. Intraspinal accumulation of circulating leukocytes, microglia, reactive oxygen species, and inflammatory mediators, such as interleukin-1β (IL-1β), TNF-α, and interleukin-6 (IL-6), are known to be markedly diminished in the chronic SCI setting, at times greater than four weeks after injury [72,73,74,75,76,77]. A comparison of iNOS localization in the chronic spinal cord, relative to the acute SCI setting, might shed light on the etiology of the worsened histological outcomes observed in our study.

Dorsal column fibers were quantified at the study’s endpoint (Figure 2A) in order to shed light on the observed regression of locomotor improvements seen after acute suppression of iNOS following SCI (Figure 3A). Fibers of interest were located in the white matter of the thoracic spinal cord, ventral to the Fasciculus Gracilis and Cuneatus, within the dorsal column region [78,79,80]. The majority of these fibers constitute the dorsal corticospinal tract (dCST), which is the dominant mouse corticospinal tract encompassing >90% of total CST axons, representing the CST most vulnerable to damage from dorsal contusive spinal cord injury [79]. This region also includes occasional passing fibers from the spinothalamic tract’s decussation as the anterior white commissure. These fibers are in the minority, relative to the ~5000 dCST axons present in this location [79]. Our method of labeling the total number of neurofilament (NF)^+^ axons in this area provides a gross estimation of preserved dCST axons, as it does not differentiate between preserved and regenerating axons. Future studies might wish to perform anterograde tracing from the motor cortex to further evaluate the role of iNOS in axonal regeneration post-SCI. Our study’s inclusion of female-only mice might have contributed to relative axonal sparing of the dCST, compared to results obtained from other studies involving the inclusion of iNOS suppression in male mice. Recently, subtle differences between the post-SCI ambulatory abilities of male and female mice have been seen, due to the differential expression of potentially neuroprotective sex hormones, such as estrogen and progesterone [81,82,83,84,85,86]. Female-only mice, as well as iNOS^−/−^ mice with the same genetic background as our WT group, were utilized to minimize intra-study variability.

In sum, our findings build upon the work from our group and others [12,27,30,31,32], which have shown that the acute antagonism of iNOS is beneficial for ameliorating deleterious inflammatory and apoptotic signaling, reducing glial reactivity and neuronal loss, in addition to improving functional recovery after SCI, by examining whether longer term, complete iNOS ablation can provide greater therapeutic efficacy. Contrary to this reasoning, we found that persistent ablation of iNOS after SCI, as has been observed in other neurological indications, worsened histopathological outcomes, and was without functional benefit, implying that such persistent iNOS ablation interfered with important processes involved in injury resolution, angiogenesis, and endogenous recovery. Notably, despite a worsening histopathology at six weeks compared to wild-type controls, iNOS knockout mice displayed no significant difference in functional outcomes. Beneficial neuroprotective actions of acute iNOS inhibition may have offset, to some degree, the latter interference of long-term iNOS inhibition on normal tissue injury resolution and functional recovery. A limitation of the present investigation was the restriction of histopathological tissue examination to the study’s endpoint, at six weeks, which restricted the interpretation of function-pathology relationships to that point in time. Our prior investigations of short-term pharmacological and antisense oligonucleotide inhibition of iNOS demonstrated a significant retardation in neuronal death both rostral and caudal to the injury site, when iNOS inhibition occurred during the first week post-injury. These results were associated with a long-term improvement in function, which suggests, in light of the current work, that an acute and temporally restricted inhibition of iNOS is needed for the provision of therapeutic benefit. Further studies which temporally examine the cell-specific role that iNOS and NO play in tissue remodeling, angiogenesis, and repair versus deleterious inflammatory processes, and neural cell death, may help to define the optimal window for future therapies that can acutely block iNOS after SCI, dampening inflammation and promoting neuroprotection, without hindering its later beneficial actions in injury resolution.

## 4. Experimental Procedures

### 4.1. Animals

Eight week old female CB57BL/6 wild-type (*iNOS^+/+^*, *n* = 17) and B6.129P2-*Nos2*^tm1Lau^ knockout mice (*iNOS*^−*/*−^, *n* = 9; The Jackson Laboratory, Bar Harbor, ME, USA), weighing 27–30 g were used for these experiments. Animals were housed according to the National Institute of Health’s guide for the Care and Use of Animals (ISBN: 0-309-15401-4, NRC 2011). The Institutional Animal Care and Use Committee of the University of Miami approved all animal procedures.

### 4.2. Moderate Thoracic Contusion Injury

Prior to surgery, mice were anesthetized (70 mg/kg ketamine and 5 mg/kg xylazine) via intraperitoneal injection. An adequate level of anesthesia was determined by monitoring the corneal reflex and stimulus-induced withdrawal of the hind limbs. The thoracic region was shaved and aseptically prepared with chlorhexidine (Phoenix Pharmaceutical, St. Joseph, MO, USA). Lacrilube ophthalmic ointment (Allergan Pharmaceuticals, Irvine, CA, USA) was applied to the eyes to prevent drying. During surgery, mice were kept on a homeothermic blanket system (Harvard Apparatus, Kent, UK) to maintain a body temperature of 37 ± 0.5 °C, as measured by a rectal probe. A rostral-caudal superficial incision was then made along the thoracic spine, followed by retraction of the skin and muscle to reveal the thoracic vertebrae. A laminectomy performed at thoracic vertebra T8 exposed the dorsal surface of the spinal cord underneath, without damaging the dura mater. Stabilization clamps were placed around the vertebrae at T9 and T10 to support the column during impact. Contusion injury was created by thoracic spinal cord displacement using the Electromagnetic SCI Device (ESCID), as previously described [87,88]. In brief, the tip of the contusion device was first lowered onto the spinal cord until a force of 2 kDyne was recorded on the force transducer. Approximately 2 s after this pre-load force was reached, contusive spinal cord injury (SCI) was created by a rapid displacement of the impact head (0.5 mm displacement, moderate injury). For the inducible nitric oxide synthase (*iNOS*)*^+/+^* wild-type (WT) mice, nine specimens underwent a moderate thoracic T8 contusive, whereas the other eight specimens were used as sham controls, receiving a laminectomy, but no SCI. All nine *iNOS^−/−^* mice received contusive SCI. Two mice, one *inos*-null and one WT, did not survive acutely post-SCI, due to complications relating to the SCI and/or anesthesia. After injury, the muscles and skin were closed in layers, using sutures. The mice were allowed to recover in a warmed cage, with readily accessible water and food. Gentamicin (5 mg/kg, intramuscular (i.m.); Abbott Laboratories, North Chicago, IL, USA) was immediately administered post-surgery and then daily, for seven days. The analgesic buprenorphine (0.1 mg/kg sub-cutaneous (s.c.); Reckitt Benckiser, Richmond, VA, USA) was delivered post-surgery and repeated daily for two days.

### 4.3. Histology

Mice were euthanized at six weeks post-SCI (100 mg/kg ketamine, 15 mg/kg xylazine) and transcardially perfused, first with saline, followed by phosphate-buffered, 4% paraformaldehyde (0.1 M, pH 7.4). The thoracic spinal cord, containing the complete lesion, was dissected and post-fixed in 4% paraformaldehyde for five days, prior to embedding in paraffin.

### 4.4. Estimation of Tissue Volumes

An 8 mm paraffin-embedded segment of spinal cord encompassing the injury site at its center, was coronally sectioned at 10 µm, into 20 series. One series of sections was stained with hematoxylin, eosin, and Luxol fast blue, for gray and white matter visualization. Sections located at every 400 µm were used for analysis of white and gray matter tissue preservation by computer-assisted microscopy and Neurolucida software (version 4.5, MicroBrightField Bioscience, Colchester, VT, USA), as described previously [78]. Preserved tissue was distinguished from damaged tissue by the absence of tissue infiltrating immune cells, intact myelin, and/or the presence of healthy appearing neurons. The presence of viable neurons and tissue-infiltrating immune cells were assessed by their respective cytomorphologic features on hematoxylin and eosin staining. The extent of myelination was qualitatively assessed by Luxol fast blue staining. The individual areas of preserved white and gray matter in each section were first independently contoured and measured by a researcher blinded to the identity of the mouse (iNOS KO or WT), from which the tissue was obtained, after which the volumes of each was determined within the injured spinal cord segment using the NeuroExplorer algorithms [81].

### 4.5. Immunohistochemistry and Quantification of Stained Profiles

To enable the evaluation of the number of preserved axons and blood vessels in perilesional regions rostral to the lesion center, 10 µm spinal cord sections (200 µm intervals) underwent immunohistochemical (IHC) staining with antibodies specific to these elements. First, sections were deparaffinized and then underwent non-specific immunoglobulin binding, blocked by incubation with 5% normal goat serum (NGS) in phosphate-buffered saline (PBS) for 1 h. Following this, blocked sections were incubated with the following primary antibodies, either alone or in combination: mouse monoclonal pan-anti-neurofilaments (1:2000; Neurofilament light (NF-L) clone MCA-DA2 and Neurofilament medium (NF-M) clone MCA-3H11 from Encor Biotechnology Inc., Gainesville, FL, USA; and Neurofilament heavy (NF-H) clone RT97 from The Developmental Studies Hybridoma Bank, Iowa City, IA, USA), mouse monoclonal anti-α-smooth muscle actin (α-SMA; 1:400; Sigma Chemical Company, St. Louis, MO, USA), rabbit polyclonal anti-glial fibrillary acidic protein (GFAP; 1:500; Dako, Carpentaria, CA, USA), and Dylight-594-labelled *Lycopersicon Esculentum* (Tomato) lectin (1:200; Vector Laboratories, Burlingame, CA, USA) in 5% Normal Goat Serum/Phosphate-buffered saline (NGS/PBS). For NF or α-SMA IHC, after an overnight primary incubation at room temperature, sections were washed (3× in PBS, 5 min each), then placed with a biotin-conjugated, goat anti-mouse secondary antibody (1:1000, Santa Cruz Biotechnology, Dallas, TX, USA) in 2% NGS/PBS, for 2 h at room temperature. Sections were then washed again (2× in PBS, 5 min each) and incubated with horse radish peroxidase-conjugated streptavidin (Vector Labs, Burlingame, CA, USA) for 1 h at room temperature. Visualization of NFs was accomplished using the VIP chromogen kit (1:100, Vector Labs; for 10 min) to yield a purple precipitate for labelling axons or the 3,3′-diaminobenzidine (DAB, 0.05%; Vector Labs; for 3 min) chromogen kit to yield a brown precipitate for identifying blood vessels. Sections were counterstained with methyl green to label nuclei. For sections incubated with the combination of GFAP and Tomato-lectin antibodies, they were first incubated with the primary antibodies overnight at 4 °C, followed by subsequent washes (3× PBS, 5 min each), dried for 5 min at 40 °C, and then cover slipped using Vectamount mounting medium (Vector Labs). For stereological counting of NF^+^ axons or α–SMA^+^ blood vessels, sections within the perilesional area rostral to the injury epicenter were analyzed using StereoInvestigator software (MicroBrightfield), by a researcher blinded to the identity of the mouse (iNOS KO or WT) from which the tissue was obtained at specific distances from the injury epicenter. For NF-stained axon profiles, the region encompassing the dorsal columns in each section, located at distances of 600, 1600, and 3000 µm from the lesion epicenter, was first contoured and then divided into grids for counting the total number of stained axon profiles at each distance, as described previously [78]. For blood vessels, in immunostained sections at 600 µm from the injury epicenter, the dorsal and lateral white matter regions were contoured in two consecutive sections per animal, and the number of blood vessels per 100 µm^2^ was quantified and averaged across sections.

### 4.6. Behavioral Testing

Hind limb performance in the open field was assessed weekly by two researchers, blinded to the animals’ genetic background, for six weeks post-SCI using the Basso Mouse Scale (BMS) for locomotion [89]. For recording the scores, mice were placed in a round plastic pool with a smooth surface and were observed for unique features of hind limb movement during a 4 min period. Hind limb movements immediately after contact with the plastic surface were disregarded. In addition to the BMS scoring, hind paw placement was evaluated during walking, using footprint analysis at the study’s endpoint (six weeks post-SCI), using a modified protocol [90,91]. The hind paws of the mice were first inked and footprints were made on a length of paper covering the floor of a narrow runway (100 cm long by 5 cm wide, which provided a standardized direction of movement). A series of at least six sequential steps was used to determine the mean value for each measurement of limb rotation, stride length, and base of support. The base of support was obtained by measuring the distance between the centers of the pads of the contralateral hind paws. Limb rotation was quantified by using the angle of the central digit deviation from a line drawn along the center of the pads of consecutive hind paw placements in the direction of walking. Stride length was measured as the distance between the centers of the pads of two consecutive footprints of the same hind paw.

### 4.7. Statistical Analysis

A one-way analysis of variance (ANOVA) with Bonferroni post-hoc test was used to compare counts of axons and blood vessels, at specific distances rostral to the injury site among groups. For analysis of weekly functional recovery patterns, a mixed factorial, repeated-measures ANOVA, followed by the Tukey-Kramer post-test, was employed. Differences were deemed statistically significant at * *p <* 0.05, ^#^ *p <* 0.05, ** *p* < 0.01, ^##^ *p* < 0.01, *** *p* < 0.001, or ^###^ *p* < 0.001; comparisons were made to either * wild-type or ^#^ sham controls. All errors are given as the standard error of the mean.
